# Permeability evaluation of gemcitabine-CPP6 conjugates in Caco-2 cells

**DOI:** 10.5599/admet.882

**Published:** 2020-10-26

**Authors:** Abigail Ferreira, Sara Moreira, Rui Lapa, Nuno Vale

**Affiliations:** 1 OncoPharma Research Group, Center for Health Technology and Services Research (CINTESIS), Rua Dr. Plácido da Costa, 4200-450 Porto, Portugal; 2 LAQV/REQUIMTE, Laboratory of Applied Chemistry, Department of Chemical Sciences, Faculty of Pharmacy, University of Porto, Rua de Jorge Viterbo Ferreira, 228, 4050-313 Porto, Portugal; 3 Faculty of Sciences, University of Porto, Rua do Campo Alegre, 687, 4169-007 Porto, Portugal; 4 Faculty of Medicine, University of Porto, Al. Prof. Hernâni Monteiro, 4200-319 Porto, Portugal

**Keywords:** Cell-Penetrating Peptides, Permeability, Solubility, Caco-2, DMPK

## Abstract

Cancer is one of the most alarming diseases due to its high mortality and still increasing incidence rate. Currently available treatments for this condition present several shortcomings and new options are continuously being developed and evaluated, aiming at increasing the overall treatment efficiency and reducing associated adverse side effects. Gemcitabine has proven activity and is used in chemotherapy. However, its therapeutic efficiency is limited by its low bioavailability as a result of rapid enzymatic inactivation. Additionally, tumor cells often develop drug resistance after initial tumor regression related to transporter deficiency. We have previously developed three gemcitabine conjugates with cell-penetrating hexapeptides (CPP6) to facilitate intracellular delivery of this drug while also preventing enzymatic deamination. The bioactivity of these new prodrugs was evaluated in different cell lines and showed promising results. Here, we assessed the absorption and permeability across Caco-2 monolayers of these conjugates in comparison with gemcitabine and the respective isolated cell-penetrating peptides (CPPs). CPP6-2 (KLPVMW) and respective Gem-CPP6-2 conjugate showed the highest permeability in Caco-2 cells.

## Introduction

Cancer is the second leading cause of death worldwide. There were 18 million new cases and 9.6 million cancer related deaths in 2018. Globally, about one in six deaths is due to cancer. Lung and prostate cancers are the most prevalent in men, while breast and colorectum cancers affect women the most [1]. Drug resistance and overall treatment inefficacy are responsible for the high mortality of this multifactorial disease. Difficulty in recognizing altered cells and distinguishing them from normal cells as well as reaching metastases are the main hurdles of cancer therapy. Research in this field strives to maximize efficacy while reducing adverse side effects.

**Scheme 1.**
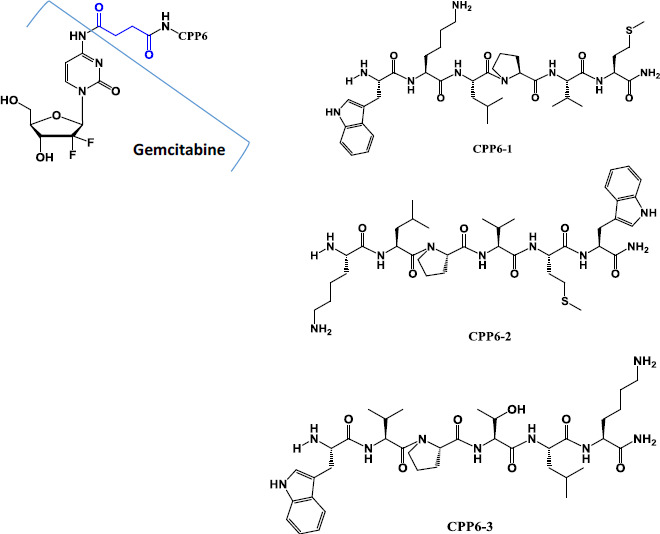
Chemical structure of gemcitabine (before and after modification and conjugation with CPP6) and of the three CPP6 studied, CPP6-1 (WKLPVM), CPP6-2 (KLPVMW) and CPP6-3 (WVPMLK).

Gemcitabine (Gem, dFdC or 2′,2′-difluoro-2′-deoxycytidine, Scheme 1) is a cytotoxic nucleoside analogue used as a chemotherapeutic drug. It is effective against an extensive range of solid tumors, such as pancreatic, non-small cell lung, breast and ovarian cancers, and is often a first-line treatment in clinical settings [2]. Despite being administered intravenously, treatment with gemcitabine has limited efficacy largely due to its short half-life (8-17 min), since gemcitabine is rapidly inactivated metabolically in the serum through deamination of its 4-N amine by cytidine deaminase (CDA), present in high levels in both human plasma and liver. As such, much higher doses are required to reach an effective plasma concentration, increasing toxicity and the risk of adverse side effects. Another major obstacle is the drug resistance related to nucleoside transporter deficiency, which is developed by some tumor cells after initial tumor regression. As a hydrophilic drug, gemcitabine cellular uptake is primarily facilitated by the human equilibrative nucleoside transporter 1 (hENT1). Thus, the expression of this transporter plays a key role in gemcitabine intracellular uptake [3]. There have been many efforts to improve gemcitabine clinical efficacy and numerous derivatives and prodrugs have been designed to alter some of the unfavorable physicochemical properties of gemcitabine and ideally improve its oral bioavailability [4]. Our research group has previously developed gemcitabine conjugates with cell-penetrating peptides (CPPs) to facilitate intracellular delivery of this drug [5-6]. In three of these new prodrugs, a cell-penetrating hexapeptide (CPP6) was covalently conjugated to the aniline moiety of gemcitabine through suitable bio-/chemo-reversible bonds. Two CPP5 (KLPVM and VPMLK) with reported high percentage of cell-penetration were selected. One tryptophan (Trp) residue was added to the terminal of these CPP5 to further improve their capacity of cell-penetration, since Trp has high propensity to be inserted into membranes [7-8], yielding three novel hexapeptides: CPP6-1 (WKLPVM), CPP6-2 (KLPVMW), and CPP6-3 (WVPMLK). These conjugates were designed to facilitate intracellular delivery of this drug and even overcome the problem of transporter deficiency related drug resistance, as CPPs are non-cytotoxic vectors and drug delivery vehicles. Additionally, gemcitabine is protected from CDA enzymatic deamination since its 4-N amine was modified. The bioactivity of these new prodrugs was previously evaluated in different cell lines and showed promising results [5].

In this work, we have evaluated the absorption and permeability of gemcitabine, three CPP6 and the respective Gem-CPP6 conjugates across Caco-2 monolayers. The single layer of epithelial cells that covers the inner intestinal wall forms the rate-limiting barrier to the absorption of dissolved compounds administered orally. Consequently, proper reconstitution of a human differentiated epithelial cell monolayer *in vitro* can be used to predict the absorption and permeability coefficients of orally administered drugs. The human colon carcinoma cell line Caco-2 has been successfully used to fulfill this purpose and monolayers grown on permeable filters have become the in vitro golden standard for these predictions [9]. This method is recognized by the American Food and Drug Administration (FDA) [10].

## Experimental

### Cell culture

Human colon adenocarcinoma cell line Caco-2 (passage 25-47, kindly provided by the Department of Biomedicine of the Faculty of Medicine of University of Porto, and previously acquired via ATCC) was routinely maintained in Dulbecco’s Modified Eagle’s Medium (DMEM) supplemented with 10 % fetal bovine serum (FBS), L-glutamine and antibiotics penicillin and streptomycin. Cells were cultured at 37 °C in a 5 % CO_2_ humidified atmosphere. Culturing medium was replaced every 2-3 days and cell subculture was conducted once a week by trypsinization (0.25 % trypsin-EDTA wt/vol, 5, min, 37, °C).

### Cytotoxicity assay

The cell growth inhibitory activity of gemcitabine in Caco-2 cells was assessed with the MTT assay. Briefly, a growth curve was traced to determine the best cell density for the assay. Cells were seeded in 96-well plates (growth surface 0.322 cm^2^, from TPP®, Product No. 92696) with an initial cell density of 9.3 x 10^3^ cells/mL (200 μL per well). Cells were allowed to attach for 24 h and were then either left untreated (culture medium was replaced for fresh medium) or treated with gemcitabine (0.1, 1, 5, 10, 20, 50, 100, 1000, 10000 and 100000 μM). Following 72 h incubation, cell medium was removed and 100 μL of MTT (3-(4,5-dimethylthiazol-2-yl)-2,5-diphenyltetrazolium bromide) solution were added per well. Cells were then incubated for another 4 h protected from light. Finally, MTT solution was removed, DMSO (100 μL per well) was added to solubilize the formazan crystals formed by viable cells and absorbance was measured at 570 nm in an automated microplate reader (Sinergy HT, Biotek Instruments Inc, Vermont, USA). All conditions were performed in triplicate.

Gemcitabine cytotoxicity results were compared with the untreated control (mean of values was set to 100 %) and expressed as mean ± SEM. The statistical significance between different gemcitabine concentrations was analyzed in GraphPad Prism 7 (San Diego, EUA) using one-way ANOVA (p<0.05).

### Preparation of CPPs and gemcitabine-CPP6 conjugates

All the compounds evaluated in this work (except for Gemcitabine, that was purchase from Sigma-Aldrich as Gemcitabine hydrochloride, G6423) had been previously synthesized by our research group [5]. Briefly, CPP6-1 (WKLPVM), CPP6-2 (KLPVMW) and CPP6-3 (WVPTLK) were manually synthesized using standard solid phase peptide synthesis (SPPS) and Fmoc chemistry. Gemcitabine was modified to allow conjugation with CPP6. First, the two hydroxyl groups of gemcitabine were selectively protected with Boc groups; then, succinic anhydride was linked to the 4-N amine and lastly a CPP6 was conjugated to this anticancer drug. Some properties of these new conjugates are presented in Table 1.

### Permeability assay

A monolayer of Caco-2 cells was established in a 12-well plate [11]. Each well contains a permeable filter insert, a transparent collagen treated (equimolar mixture of types I and III collagen) polytetrafluoroethylene (PTFE) membrane, with 12 mm diameter and 0.4 μm pore (Corning Inc, Corning Transwell COL collagen coated membrane inserts, NY, USA, Cat. No. 3493). Firstly, filters were pre-wet with 0.1 mL of culture medium for 2 minutes. Cells were seeded in the apical side (0.5 mL of cells per well, cell density of 4.0 x 10^5^ cells/mL). The basolateral compartment was filled with 1.5 mL of culture medium and plates were incubated for 6 h (5 % CO_2_ humidified atmosphere at 37 °C). Then, to remove non-adherent cells and reduce the risk of multilayer formation, the medium in the apical side was removed and replaced with fresh medium. Cells were maintained for 29 days, replacing the culture medium from both compartments every other day.

### Transport across Caco-2 monolayer assay

All the solutions used were pre-warmed to 37 °C. Culture medium was replaced with fresh medium 24 h prior the beginning of the experiment. Next, this culture medium was removed first from the basolateral and then from the apical compartment. The apical compartment was carefully washed and then filled with 0.5 mL of Hank’s Balanced Salt Solution (HBSS, pH 7.4) and the basolateral compartment was also filled with 1.5 mL of HBSS. The plates were incubated for 17 min at 37 °C under gentle shaking (190 rpm, GFL® Orbital Shaker 3015). All tested compounds were prepared in HBBS (60 μM) and added to the apical compartment. Throughout this assay, the final volume was 0.4 mL in the apical compartment and 1.2 mL in the basolateral chamber. At t = 0 min, 0.45 mL of the donor solution was added to the apical compartment and a sample (0.05 mL) was immediately taken. The plate was incubated (lid-covered) at 37 °C under gentle shaking (190 rpm). Every 30 min for the next 2 h, a sample of 0.6 mL was taken from the basolateral compartment and replaced with the same volume of HBSS. After 120 min, a sample of 0.05 mL was taken from the apical side.

### HPLC quantification

The concentrations of the evaluated compound in the basolateral and apical compartments were determined by high-performance liquid chromatography (HPLC) (VWR International LCC, LaChrom Ultra, Pennsylvania, USA). Elution was performed with a variable gradient of acetonitrile (ACN) in water containing 0.05 % trifluoroacetic acid (TFA), at a 0.7 mL/min flow and detection at variable wavelength (243 nm for gemcitabine and 220 nm for peptides and conjugates). All chemicals were either analytical or HPLC grade.

## Results and discussion

### Gemcitabine cytotoxicity

**Figure 1.**
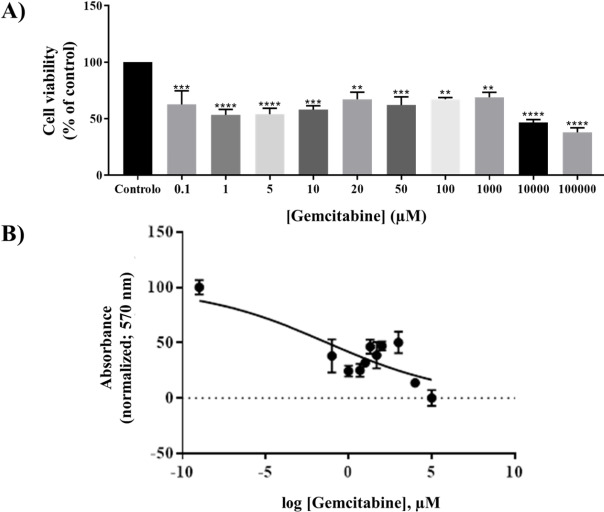
Concentration-dependent cytotoxicity of gemcitabine in Caco-2 cells (Abs 570 nm). Results are presented as (**A**) the percentage of inhibition compared to control untreated cells and (**B**) plotted as the log [gemcitabine] against the percentage of control untreated cells. Results are expressed as mean ± SEM (n=3; p<0.05, one-way ANOVA).

Every concentration of gemcitabine tested caused a statistically significant inhibition of cell growth in Caco-2 cells compared to untreated cells (Figure 1). The maximum inhibition was observed after treatment with the highest concentration of gemcitabine evaluated (100000 μM). The IC_50_ was calculated as 52.4 μM.

### Caco-2 monolayer and permeability of Gemcitabine

Microscopic analysis of the filter revealed a uniformly formed monolayer, with no detection of anomalies or areas without cells. A Caco-2 monolayer was successfully established and this is a reliable indicator for proceeding with the permeability study.

After 30 minutes, just 3 % of Gemcitabine was found in the basolateral side. At the 90 min time point, a plateau was reached and only an additional 1 % of the drug crossed the membrane until the end of assay (120 min), with a total of 18 % of the amount of Gemcitabine initially applied in the apical side having crossed into the basolateral compartment.

### Permeability of CPP6

**Figure 2.**
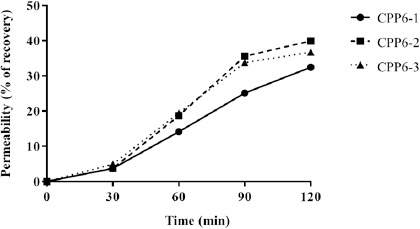
Concentration of CPP6 in the basolateral compartment after incubation with 60 μM in the apical compartment and fitted curves.

Comparable amounts of the 3 CPP6 crossed the monolayer of Caco-2 cells after 120 minutes. Still, CPP6-2 exhibited the highest permeability, with 40 % being recovered in the basolateral chamber, followed by CPP6-3 (37 %) and CPP6-1 (33 %). During the first 30 minutes, only 4 to 5 % of all CPPs reached the basolateral side; the rate of permeation was highest between 30 and 90 minutes. CPP6-2 and CPP6-3 registered a decrease in this rate for the last 30 minutes of this assay. Throughout the experiment, a slower rate of permeation was observed for CPP6-1 (Figure 2).

By the end of the experiment, the sum of the CPP absorbed from the apical side and found in the basolateral chamber was 84 % for CPP6-2, 75 % for CPP6-3 and 50 % for CPP6-1. This indicates that some peptide is retained inside the cells or was degraded.

### Permeability of Gem-CPP6 conjugates

**Figure 3.**
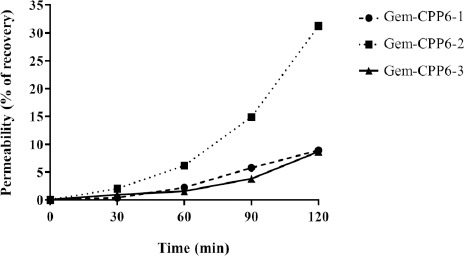
Permeability of the Gem-CPP6 conjugates, expressed as the percentage recovered in the basolateral compartment.

As for the Gem-CPP6 conjugates, the conjugate of gemcitabine with CPP6-2 clearly stands out as the most permeable across the Caco-2 cell monolayer. The extent of permeation of this conjugate was about 3-fold the observed for the Gem-CPP6-1 and Gem-CPP6-3 conjugates. Approximately 31 % of the Gem-CPP6-2 conjugate was found in the basolateral chamber, while only 9 % of Gem-CPP6-1 and Gem-CPP6-3 conjugates were able to cross the monolayer of Caco-2 cells and was quantified in this compartment (Figure 3).

The higher permeability of the Gem-CPP6-2 conjugate is in agreement with the previous evaluation of the permeability of the isolated CPP6, with CPP6-2 being the most permeable peptide. Of the three studied CPP6, CPP6-2 is the only one that has a Trp residue in its C-terminal (CPP6-1 and CPP6-3 have a Trp residue in the N-terminal position). Additionally, the only difference between CPP6-1 and CPP6-2 is the position of this amino acid residue (N-terminal versus C-terminal).

Tryptophan can be considered hydrophobic due to its uncharged side chain. Given its aromatic character, it can form hydrogen bonds and strongly interact with cellular membranes, disrupting the stability of the membrane lipidic chains. These properties grant this amino acid residue a great tendency to insert into membranes [12-15]. It has been shown that the replacement of the 2 Trp residues of the known CPP Penetratin for phenylalanine residues completely eliminates the penetration ability of this peptide [16]. Furthermore, Rydberg et al. have reported that the number of Trp residues in a CPP is relevant, with a higher number of Trp residues in a CPP corresponding to greater penetration ability [12]. These authors have also demonstrated that the position of this residue is another factor influencing permeability; in this case, CPPs with Trp residues in the N-terminal position were less absorbed.

Some properties of these new Gem-CPP6 conjugates were predicted using in silico tools and are presented in Table 1. Gem-CPP6-2 has the highest calculated P_app_ and is also predicted as the most permeable conjugate by GastroPlus™, a mechanistically based pharmacokinetics and pharmacodynamics simulation software.

## Conclusions

The monolayer of Caco-2 cells was a suitable method to study the permeability of the anticancer drug gemcitabine, the cell-penetrating hexapeptides (CPP6) and the new prodrug conjugates Gem-CPP6. Every CPP6 and Gem-CPP6 conjugate was able to cross this monolayer, suggesting their potential as drug delivery systems. In agreement with previous reports, the position of the Trp residue was a determinant factor influencing the permeability of the CPP6 and Gem-CPP6 conjugates. Amongst the evaluated compounds, CPP6-2 and the respective Gem-CPP6-2 conjugate showed the highest permeability, crossing the cell monolayer to a greater extent. This data puts forward this conjugate as a lead for further studies and as the most promising for potential clinical application.

## Figures and Tables

**Table 1.** Physicochemical and in silico pharmacokinetic data of CPP6-Gemcitabine conjugates.

Peptide/Conjugate	*M*_W_ (g/mol)	PSA ^a^ (Å^2^)	HBD	*P*_eff_ (cm/s) ^b^ (x10^-6^)	*P*_eff_ (cm/s) ^c^ (x10^-9^)	*P*_app_ (cm/s) (x10^-8^)
Gem-CPP6-1	1117.29	247.63	13	3.19	1.31	1.326
Gem-CPP6-2	1117.29	247.63	13	3.82	1.31	4.568
Gem-CPP6-3	1087.20	267.86	14	2.47	0.412	1.326

^a^ Polar surface area, predicted by MedChem Designer (version 3.1.0.30, Simulations Plus, Inc., Lancaster, California, USA);

^b^ Effective permeability, predicted by GastroPlus™ (version 9.5, Simulations Plus, Inc., Lancaster, California, USA);

^c^ Effective permeability, calculated from Winiwarter equation: Log P_eff_ = (-2.546) – (0.011 x PSA) – (0.278 x HBD); HBD: hydrogen bond donors; [17].
